# Decryption speed up of ElGamal with composite modulus

**DOI:** 10.1371/journal.pone.0240248

**Published:** 2020-10-05

**Authors:** GyuChol Kim, SuChol Li

**Affiliations:** College of Information Science and Technology, Kim Chaek University of Technology, Pyongyang, DPR of Korea; Wuhan University, CHINA

## Abstract

Public key cryptosystems such as RSA, rebalanced RSA and ElGamal have the disadvantage of serious asymmetry between encryption and decryption speed. We reduced the CRT (Chinese Remainder Theorem) exponents maintaining full sized private exponent in ElGamal with composite modulus (CRT–ElGamal) for the fast decryption as in rebalanced RSA. In this case, unlike rebalanced RSA, decryption speed up can be obtained without losing of the fast encryption speed which is comparable to RSA with small public exponent. As a result, it is possible to propose the fast public key cryptosystem in which both encryption and decryption are fast, by reducing the asymmetry (i.e., fast encryption/slow decryption) in CRT–ElGamal encryption.

## 1. Introduction

The security of ElGamal public key cryptosystem [1] depends on the intractability of the DL (Discrete Logarithm) problem. In other words, computational security of ElGamal depends on the CDH (Computational Diffie–Hellman) assumption [2] and semantic security under the passive attack depends on the DDH (Decision Diffie–Hellman) assumption [3] or HDH (Hashed Diffie–Hellman) assumption [4].

Under the DDH assumption, ElGamal is secure in the sense of indistinguishability against chosen plaintext attack (IND–CPA) and some variants of ElGamal such as Cramer-Shoup [5] are secure in the sense of indistinguishability against chosen ciphertext attack (IND–CCA).

If CDH assumption is broken then DDH and HDH assumptions will also be broken. However, CDH by itself is not sufficient to prove that ElGamal encryption is useful for practical cryptographic purpose, because it does not consider the partial exposure of plaintext. Hence, DDH assumption was proposed for the semantic security of ElGamal.

Meanwhile, using DH value itself to mask plaintext via multiplication is not recommended in practical ElGamal systems and it is recommended to hash DH value in order to obtain pseudorandom key (which is used as a one-time pad) of suitable length which can then be used to encrypt the plaintext under the semantically secure symmetric encryption (e.g., symmetric authenticated encryption).

If hash function is modeled as a random oracle, it is possible to obtain the pseudorandom key based on the CDH assumption in the non-DDH groups (i.e., the groups in which DDH assumption does not hold).

In DDH-group (i.e., the group in which DDH assumption holds), from the property of DDH assumption, it is needless for the hash function to be modeled as a random oracle (i.e., there is no need to use the random oracle).

HDH assumption which is a weaker assumption than DDH was studied [4] to get the pseudorandom key without random oracle in the non-DDH groups.

Then, what is the basis of CDH, DDH and HDH assumptions?

As in all the other public key systems, assumption for the one–way function is used as a base assumption of security in ElGamal, too.

That is, cryptographers developed the one way function

$$F(x)=g^{x}\in G\;:\;x\in K\;(K\;is\;a\;key\;space)$$ (1)

in a finite group *G* with generator *g* and, on the basis of the assumption that *F* is a one–way function, they considered the CDH, DDH and HDH assumptions and designed the ElGamal encryption protocols in various groups.

For the proof of one–wayness of *F*(*x*), assumption that DLP (Discrete Logarithm Problem) is hard (i.e., DL assumption) has been usually used, but other assumptions (e.g., DLSE (Discrete Logarithm with Short Exponents) assumption [4, 6–8]) which have been known to be hard as a DL assumption can also be used instead of DL assumption.

We proposed the one–way function based on RDL (Restricted DL with small CRT exponents) assumption, considered the various assumptions such as RCDH, RDDH and RHDH assumptions (described later) and designed the fast ElGamal encryption protocols.

First, we considered the CRT–ElGamal which is known to be semantic secure under the passive attack (i.e., which is known to be IND-CPA).

Let *G* is a multiplicative subgroup of $Z_{n(=pq)}^{*}$ with order $\lambda =\frac{\left(p-1)(q-1\right)}{2}$ where $p,q,\frac{p-1}{2}$ and $\frac{q-1}{2}$ are the prime numbers. And let *K* = *Z*_*n*_,*x*_*p*_ = *x mod* (*p*−1),*x*_*q*_ = *x mod* (*q*−1) and *H*(*G*^2^→*K*_*s*_) is a hash function, where *K*_*s*_ is a key space of symmetric authenticated encryption (*E*_*s*_, *D*_*s*_).

Then, referring to [3, 4] and [9], the following four assumptions hold for the group *G*.

### DL assumption

There is no probabilistic polynomial time algorithm *A* such that

$$\mathrm{Pr}\left[A(g,F(x))=x|x\in Z_{n}\right]>negligible.$$

DL assumption is based on the Fact 3.78 and 3.79 of [9] and from DL assumption, *F*(*x*) becomes a one–way function when *K* = *Z*_*n*_.

### CDH assumption

There is no probabilistic polynomial time algorithm *A* such that

$$\mathrm{Pr}\left[A(g,F(x),F(y))=F(xy)|x,y\in Z_{n}\right]>negligible.$$

CDH assumption is based on the assumption that *F*(*x*) is a one–way function for *x*∈*Z*_*n*_.

### DDH assumption

There is no probabilistic polynomial time algorithm *A* such that

$$\mathrm{Pr}\left\{|\mathrm{Pr}\left[A(g,F(x),F(y),F(xy))=1\right]-\mathrm{Pr}\left[A(g,F(x),F(y),F(z))=1\right]|>negligible\right|x,y,z\in Z_{n}\}>neligible$$

DDH assumption is based on the CDH assumption and the hardness of distinguishing quadratic residues from non–residues in *Z*_*n*_ [3].

### HDH assumption

There is no probabilistic polynomial time algorithm *A* such that

$$Pr\{|\mathrm{Pr}\left[A(g,F(x),F(y),H(F(y),F(xy)))=1\right]-\mathrm{Pr}\left[A(g,F(x),F(y),R)=1\right]|>negligible|x,y\in Z_{n},R\in K_{s}\}>neligible.$$

HDH assumption relies on the CDH assumption and the existence of large order DDH subgroup of *G* [4].

For the assumptions mentioned above,

DL⟸CDH⟸HDH⟸DDH

is satisfied, where “*X*⟸*Y*” denotes that assumption *X* always holds if assumption *Y* holds.

Under the assumptions above, practical CRT–ElGamal can be described as follows.

**Algorithm 1.1: Key generation for CRT-ElGamal**.

Each user creates the public key and the corresponding private key.

**Step 1.** Select a large composite number $n(=pq:p,q,\frac{p-1}{2}$ and $\frac{q-1}{2}$ are large primes) and a generator *g* of group *G*. *G* is a multiplicative subgroup of $Z_{n}^{*}$ and order of *G* is $\lambda (=\frac{\left(p-1)(q-1\right)}{2})$.

This can be described in detail as follows.

**Step 1.1.** Select the large primes *p*,*q*,*p*′ and *q*′ such that *p* = 2*p*′+1 and *q* = 2*q*′+1 and calculate *n* = *pq* and λ = *lcm*(*p*−1,*q*−1) = 2*p*′*q*′.

**Step 1.2.** Select a generator *g*_*p*_ of $Z_{p}^{*}$ and generator *g*_*q*_ of $Z_{q}^{*}$ and calculate *g* that satisfies *g*_*p*_ = *g mod p* and *g*_*q*_ = *g mod q* as follows.

$$g=\left((\left(g_{p}-g_{q})(q^{-1}mod\;p\right))mod\;p\right)q+g_{q}$$

In this case, *g* becomes a generator of subgroup *G* with order *λ*, which is the multiplicative subgroup of *Z*_*n*_.

**Step 2.** Select a random integer *x*(1≤*x*<*λ*,*gcd*(*x*,*λ*) = 1) and compute *k* = *g*^*x*^*mod n*.

This can be described in detail as follows.

**Step 2.1.** Select a random integer *x*_*p*_(1<*x*_*p*_<*p*−1) and *x*_*q*_(1<*x*_*q*_<*q*−1) such that *gcd*(*x*_*p*_,*p*−1) = 1 and *gcd*(*x*_*q*_,*q*−1) = 1. In this case, *x*_*p*_≡*x*_*q*_ *mod* 2 is satisfied.

**Step 2.2.** Calculate $k_{p}=g_{p}^{x_{p}}mod\;p,k_{q}=g_{q}^{x_{q}}mod\;q$ and

$$k=\left((\left(k_{p}-k_{q})(q^{-1}mod\;p\right))mod\;p\right)q+k_{q}$$

In this case, *k* = *g*^*x*^*mod n*,*x*_*p*_ = *x mod* (*p*−1) and *x*_*q*_ = *x mod*(*q*−1) are satisfied.

**Step 3.** Public key is (*g*,*k*,*n*) and private key is *x*.

This can be described in detail as follows.

**Step 3.1.** Public key is (*g*,*k*,*n*) and private key is (*x*,*x*_*p*_,*x*_*q*_,*p*,*q*).

For the semantic security, encryption and decryption use the symmetric authenticated encryption (*E*_*s*_,*D*_*s*_) defined over (*K*_*s*_,*M*_*s*_,*C*_*s*_) and hash function *H*(*G*^2^→*K*_*s*_).

**Algorithm 1.2: Encryption for CRT-ElGamal**.

User encrypts a message *m*∈*M*_*s*_, where *M*_*s*_ is a plaintext space of (*E*_*s*_,*D*_*s*_).

**Step 1.** Obtain authentic public key (*g*,*k*,*n*).

**Step 2.** Select a random integer *y*(1<*y*<*n*) and compute *u* = *g*^*y*^ *mod n*,*v = k*^*y*^ *mod n* and *k*_*s*_ = *H*(*u*,*v*).

**Step 3.** Encrypt the message *m* by using symmetric encryption *E*_*s*_ and key *k*_*s*_.

$$c=E_{s}(k_{s},m)$$

**Step 4.** Send the cipher text (*u*∈*G*,*c*∈*C*_*s*_). *C*_*s*_ is a cipher text space of (*E*_*s*_,*D*_*s*_).

**Algorithm 1.3: Decryption for CRT-ElGamal**.

User recovers plaintext *m* from (*u*,*c*).

**Step 1.** Compute *v* = *u*^*x*^ *mod n* and *k*_*s*_ = *H*(*u*,*v*).

**Step 1.1.** Compute *u*_*p*_ = *u mod p* and *u*_*q*_ = *u mod q*.

**Step 1.2.** Compute

$$v_{p}=u_{p}^{x_{p}}mod\;p$$

and

$$v_{q}=u_{q}^{x_{q}}mod\;q$$

**Step 1.3.** Compute *v* as follows.

$$v=\left((\left(v_{p}-v_{q})(q^{-1}mod\;p\right))mod\;p\right)q+v_{q}$$

**Step 1.4.** Compute *k*_*s*_ = *H*(*u*,*v*).

**Step 2.** Recover the message *m* by using symmetric decryption *D*_*s*_ and key *k*_*s*_.

$$m=D_{s}(k_{s},c)$$

As in CRT–RSA [10], CRT–ElGamal has an advantage to increase the decryption speed by using CRT. As mentioned above, hash function *H*(*G*^2^→*K*_*s*_) is used to extract the pseudo randomness present in the DH value and it is not necessary that *H* should be modeled as a random oracle in CRT-ElGamal because DDH assumption holds [3] in group *G*.

Next, we considered the new one–way function based on the RDL assumption and considered the RCDH, RDDH and RHDH assumptions.

Let *I*(⊆*Z*_*n*_) is a set of *x* such that ${log}_{n}x_{p}\approx {log}_{n}x_{q}\approx \delta (0<\delta <\frac{1}{2}),\;{log}_{n}x\approx 1$ and *gcd*(*x*,*λ*) = 1.

If *δ* is large enough, following RDL assumption holds. (*δ* which breaks the RDL assumption is described as *w* in Proposition 1 and 2 of Section 3.2.)

### RDL assumption

There is no probabilistic polynomial time algorithm *A* such that

Pr[A(g,F(x))=x|x∈I]>negligible.

From the RDL assumption, *F*(*x*) becomes one–way function for *x*∈*I*. In other words, the one–wayness of *F*(*x*) is not broken, even if *K* is changed from *Z*_*n*_ to *I* in Eq (1).

Hence, RCDH (Restricted CDH) assumption can be considered as follows.

### RCDH assumption

There is no probabilistic polynomial time algorithm *A* such that

$$\mathrm{Pr}\left[A(g,F(x),F(y))=F(xy)|x\in I,y\in Z_{n}\right]>negligible.$$

From DL and RDL assumptions, DDL (Decision DL) assumption can be considered as follows. (See Proposition 3 of Section 3.2 for more details.)

### DDL assumption

There is no probabilistic polynomial time algorithm *A* such that

$$\mathrm{Pr}\left\{|\mathrm{Pr}\left[A(g,F(x),I)=1\right]-\mathrm{Pr}\left[A(g,F(y),I)=1\right]|>negligible|x\in I,y\in Z_{n}\right\}>neglibible.$$

From RCDH, DDL assumptions and the hardness of distinguishing quadratic residues from non–residues [3], RHDH (Restricted HDH) assumption can be considered as follows. (See Proposition4 of Section 3.2 for more details.)

### RHDH assumption

There is no probabilistic polynomial time algorithm *A* such that

$$Pr\{|\mathrm{Pr}\left[A(g,F(x),F(y),H(F(y),F(xy)))=1\right]-\mathrm{Pr}\left[A(g,F(x),F(y),R)=1\right]|>negligible|x\in I,y\in Z_{n},R\in K_{s}\}>neligible.$$

From RCDH, DDL and DDH assumptions, RDDH (Restricted DDH) assumption can be considered as follows. (See Proposition 5 of Section 3.2 for more details.)

### RDDH assumption

There is no probabilistic polynomial time algorithm *A* such that

$$\mathrm{Pr}\left\{|\mathrm{Pr}\left[A(g,F(x),F(y),F(xy))=1\right]-\mathrm{Pr}\left[A(g,F(x),F(y),F(z))=1\right]|>negligible\right|x\in I,y,z\in Z_{n}\}>neligible.$$

From the definitions above, it can be seen that

RDL,DL⟸RCDH,DDL⟸RHDH⟸RDDH

is satisfied.

Lastly, on the basis of RCDH, RDDH and RHDH assumptions, we described the possibility of reducing CRT private exponents in the CRT–ElGamal key generation for the fast decryption.

Unlike rebalanced RSA, in this case, encryption speed is not affected. (Practically, encryption of ElGamal can be done fast [9, Section 8.4.1] by using the pre-calculated table that contains the main exponentiations of generator and public key and random exponents with low Hamming weights).

As a result, it is possible to make both encryption and decryption fast by reducing CRT exponents in CRT–ElGamal.

This paper is organized as follows. In Section 2, we reviewed the rebalanced RSA briefly. In Section 3, we described the possibility of reducing CRT exponents in CRT–ElGamal. In Section 4, we presented the theoretical and experimental results. In Section 5, we mentioned the possibility of decryption speed up in the other variants of ElGamal such as twin ElGamal [11] and Cramer-Shoup scheme [5]. Finally we concluded this paper in Section 6.

## 2. RSA assumption and rebalanced RSA

The security of RSA [12] public key encryption depends on the intractability of the IFP (Integer Factorization Problem). More precisely, computational security of RSA depends on the RSA assumption. However, as in the other public key cryptosystems, the base assumption is the assumption for the one–way function in RSA, too.

Let *n* = *pq*,*λ* = *lcm*(*p*−1,*q*−1),*d*_*p*_ = *d mod* (*p*−1) and *d*_*q*_ = *d mod* (*q*−1) where *p* and *q* are primes. Cryptographers developed the one way function

$$F(x)=x^{e}mod\;n:x\in Z_{n}^{*},e=d^{-1}\;mod\;\lambda ,d\in K\;(K\;is\;a\;key\;space)$$ (2)

under the assumption that IFP is hard (i.e., IF assumption) and, on the basis of the assumption that *F* is a one–way function, considered the RSA assumption and designed the practical RSA protocols such as RSA–OAEP [13].

Let *I* is a set of *d* such that ${log}_{n}d_{p}\approx {log}_{n}d_{q}\approx \frac{1}{2},\;{log}_{n}d\approx 1$ and *gcd*(*d*,*λ*) = 1. Further let *J* is a set of *d* such that ${log}_{n}d_{p}\approx {log}_{n}d_{q}\approx \delta (0<\delta <\frac{1}{2}),\;{log}_{n}d\approx 1$ and *gcd*(*d*,*λ*) = 1.

When *K* = *I* or *K* = *J*, *F*(*x*) of Eq (2) becomes the one–way function [14–25] and from this, following RSA assumption holds.

### RSA assumption

There is no probabilistic polynomial time algorithm *A* such that

$$\mathrm{Pr}\left[A(n,e,F(x))=x|x\in Z_{n}^{*}\right]>negligible.$$

However, RSA assumption is not sufficient to prove that RSA is useful for the practical cryptographic purpose, because it does not provide the semantic security.

Hence, RSA is usually used with symmetric authenticated encryption or padding scheme (RSA-OAEP). In both cases, RSA has been believed to be semantic secure under the RSA assumption.

For the convenience of comparison in Section 4, we considered the RSA with symmetric authenticated encryption (We simply called this CRT–RSA later on.) in detail as follows.

**Algorithm 2.1: Key generation for CRT–RSA**.

**Step 1.** Select a large composite number *n*(= *pq*:*p* and *q* are large primes) and compute *λ* = *lcm*(*p*−1,*q*−1).

**Step 2.** Select a random integer *e*(1<*e*<*λ*) such that *gcd*(*e*,*λ*) = 1.

**Step 3.** Compute *d* = *e*^−1^*mod λ*, *d*_*p*_ = *d mod*(*p*−1) and *d*_*q*_ = *d mod*(*q*−1).

**Step 4.** Public key is (*n*,*e*) and private key is (*p*,*q*,*d*,*d*_*p*_,*d*_*q*_).

For the semantic security, encryption and decryption use the symmetric authenticated encryption (*E*_*s*_,*D*_*s*_) defined over (*K*_*s*_,*M*_*s*_,*C*_*s*_) and hash function *H*(*Z*_*n*_→*K*_*s*_).

**Algorithm 2.2: Encryption for CRT–RSA**.

User encrypts a message *m*∈*M*_*s*_, where *M*_*s*_ is a plaintext space of (*E*_*s*_,*D*_*s*_).

**Step 1.** Obtain authentic public key (*n*,*e*).

**Step 2.** Choose a random integer *x* in $Z_{n}^{*}$ and compute *y* = *x*^*e*^ *mod n* and *k*_*s*_ = *H*(*x*).

**Step 3.** Encrypt the message *m* by using symmetric encryption *E*_*s*_ and key *k*_*s*_.

$$c=E_{s}(k_{s},m)$$

**Step 4.** Send the cipher text $\left(y\in Z_{n}^{*},c\in C_{s}\right)$. *C*_*s*_ is a cipher text space of (*E*_*s*_,*D*_*s*_).

**Algorithm 2.3: Decryption for CRT–RSA**.

User recovers plaintext *m* from (*y*,*c*).

**Step 1.** Compute *x* = *y*^*d*^ *mod n* and *k*_*s*_ = *H*(*x*).

**Step 1.1.** Compute *y*_*p*_ = *y mod p* and *y*_*q*_ = *y mod q*.

**Step 1.2.** Compute

$$x_{p}=y_{p}^{d_{p}}mod\;p$$

and

$$x_{q}=y_{q}^{d_{q}}mod\;q$$

**Step 1.3.** Compute *x* as follows.

$$x=\left((\left(x_{p}-x_{q})(q^{-1}mod\;p\right))mod\;p\right)q+x_{q}$$

**Step 1.4.** Compute *k*_*s*_ = *H*(*x*).

**Step 2.** Recover the message *m* by using symmetric decryption *D*_*s*_ and key *k*_*s*_.

$$m=D_{s}(k_{s},c)$$

If *H* is modeled as a random oracle, CRT–RSA is believed to be semantic secure under RSA assumption.

When *K* = *J*, RSA is called rebalanced RSA [17]. That is, rebalanced RSA is a variant that changes the key generation in RSA for the fast decryption (or signature generation).

The main issue of rebalanced RSA is to reduce the private CRT exponents *d*_*p*_ and *d*_*q*_ while maintaining private exponent *d* of the same bit size as modulus *n*.

For the security proof of proposed scheme, we considered the case that *p* and *q* are the safe primes (i.e., $\frac{p-1}{2}$ and $\frac{q-1}{2}$ are the primes).

Of course, such a restriction does not compromise the security of RSA and rebalanced RSA (i.e., RSA and rebalanced RSA with modulus *n* = (2*p*′+1)(2*q*′+1) where *p*′ and *q*′ are primes have been believed to be secure). In this case, the key generation of rebalanced RSA can be described as follows.

**Algorithm 2.4: Key generation for the rebalanced RSA using safe primes**.

**Step 1.** Select a large composite number $n(=pq:p,q,\frac{p-1}{2}$ and $\frac{q-1}{2}$ are large primes) and compute *λ* = *lcm*(*p*−1,*q*−1).

**Step 2.** Pick two random *w*(*w*<1/2*log*_2_*n*)–bit values *d*_*p*_ and *d*_*q*_ such that *gcd*(*d*_*p*_,*p*−1) = 1,*gcd*(*d*_*q*_,*q*−1) = 1 and *d*_*p*_≡*d*_*q*_*mod* 2.

**Step 3.** Find *d* such that *d* = *d*_*p*_*mod*(*p*−1) and *d* = *d*_*q*_*mod*(*q*−1).

**Step 4.** Compute *e* = *d*^−1^*mod λ*.

**Step 5.** Public key is (*n*,*e*) and private key is (*p*,*q*,*d*,*d*_*p*_,*d*_*q*_).

In rebalanced RSA, *d*_*p*_ and *d*_*q*_ are small and so, decryption can be done faster than CRT–RSA.

However, *e* will increase to be of the same bit size as modulus *n* and it will cause encryption (or signature verification) speed to be further slowed down [17] compared to standard CRT–RSA that uses 3 or 65537 as public exponent *e*.

The representative small CRT exponent attacks to rebalanced RSA were introduced in many references such as [17–25] and so on. Among them, both lattice based attack [18–25] and continued fraction attack [18] are related to the bit size of public exponent *e* and only the attack of [17, Section 4] is not related the bit size of *e*. Meanwhile, in rebalanced RSA, unless prime generation is not modified (See [18, 20] and [21]), public exponent *e* is usually full sized (*log*_*n*_*e*≈1) and so, only the attacks of [17, 20, 23] and [24] can be applied.

When *log*_*n*_*e*≈1, attack of [24, Theorem5] works for *δ*<0.122<1/2−1/7 (*min*(*d*_*p*_,*d*_*q*_)<2^250^ for the 2048bits modulus *n*), which is better than [20, Section 4](*δ*<0.073) and [17, Section 4] (*min*(*d*_*p*_,*d*_*q*_)<2^224^ for the 2048bits modulus *n*). From this, *min*(*d*_*p*_,*d*_*q*_)≥2^250^(*w*≥250) must be satisfied for the 2048bits modulus in order that RSA assumption still holds in rebalanced RSA (in order for the state-of-the-art small CRT exponent attack of [24, Theorem5] to match the current estimated complexity of factoring modulus).

In other words, rebalanced RSA (i.e, RSA (250, 250, 2048)) becomes to be (*t*,*ε*) secure under the assumption that CRT–RSA (1024, 1024, 2048) is (*t*,*ε*) secure, in which the triple indicates the bit length of CRT exponents *d*_*p*_,*d*_*q*_ and modulus *n*.

Note. We say that RSA is (*t*,*ε*) secure if no *t*-time algorithm has advantage *ε* in finding plaintext from public key and ciphertext. This is the strict definition for the computational security of RSA and in this way, the computational security of other public key cryptosystems can be redefined more strictly.

Since RSA assumption still holds in rebalanced RSA, both rebalanced RSA with symmetric authenticated encryption and rebalanced RSA with OAEP become to be semantically secure.

From all facts above, it can be seen that even though CRT exponents are reduced, CRT–RSA is still semantically secure unless the computational security is not broken.

Meanwhile, in the CRT–ElGamal, when the CRT exponents are reduced, many problems except for the computational security have to be considered, unlike the case of CRT–RSA. We considered about this in detail as follows.

## 3. Possibility of fast decryption in CRT-ElGamal

In RSA, possibility to use the small private exponent instead of full-length exponent has been introduced with small private exponent attacks [14–16, 22]. However, RSA with small private exponents has not been usually used in practical applications. Similarly, possibility to replace the full-length exponent with shorter exponent has been introduced [4, Section 4, 6–8] with DLSE assumption in ElGamal, but ElGamal with short exponents also has not been widely used in practical applications, as RSA with small private exponents.

As a result, it is not recommended to use the small private exponent instead of full-length exponent for the practical cryptographic purpose in both RSA and ElGamal.

In practical RSA applications, the scheme that reduces the CRT exponents *d*_*p*_ and *d*_*q*_ instead of *d* (i.e., rebalanced RSA) has been used for the fast decryption. In practical ElGamal applications, secure groups with small order (e.g., prime order subgroup of $Z_{p}^{*}$) have been used for the fast decryption.

As in rebalanced RSA, it would be possible to propose the fast ElGamal scheme by reducing the CRT exponents *x*_*p*_ and *x*_*q*_ instead of *x* in CRT–ElGamal. In this case, decryption can be done faster (mentioned in Section 4) than ElGamal in subgroup of $Z_{p}^{*}$, which is currently used.

We described the possibility of reducing CRT exponents in CRT-ElGamal and set the reduction bound (noted as *w* in this section) of CRT exponents *x*_*p*_ and *x*_*q*_.

### 3.1 Reducing the CRT exponents in CRT-ElGamal key generation

Key generation algorithm of proposed scheme can be described similarly to Algorithm 1.1.

Compared to key generation of CRT–ElGamal (Algorithm1.1), the selection range of *x*_*p*_(*x*_*q*_) is only reduced to 2^*w*^ from *p*−1(or *q*−1) in step 2.1. The reduction bound *w*(<1/2*log*_2_*n*) is discussed in later, so skipped here.

That is, the key generation algorithm of proposed scheme is same as the one of the CRT–ElGamal except for the Step 2.1, which can be described as follows.

**Step 2.1.** Select two random *w*(*w*<1/2*log*_2_*n*)–bit integers *x*_*p*_ and *x*_*q*_ such that *gcd*(*x*_*p*_,*p*−1) = 1, *gcd*(*x*_*q*_,*q*−1) = 1 and *x*_*p*_≡*x*_*q*_*mod* 2.

And key generation algorithm of proposed scheme can also be described similarly to Algorithm 2.4 as follows.

**Algorithm 3.1: Key generation for the proposed scheme**.

**Step 1.** Select a large composite number $n(=pq:p,q,\frac{p-1}{2}$ and $\frac{q-1}{2}$ are large primes) and calculate *λ* = *lcm*(*p*−1,*q*−1).

**Step 2.** Pick two random *w*(*w*<1/2*log*_2_*n*)–bit values *x*_*p*_ and *x*_*q*_ such that *gcd*(*x*_*p*_,*p*−1) = 1,*gcd*(*x*_*q*_,*q*−1) = 1 and *x*_*p*_≡*x*_*q*_*mod* 2.

**Step 3.** Find a *x* such that *x* = *x*_*p*_*mod*(*p*−1) and *x* = *x*_*q*_*mod*(*q*−1).

**Step 4.** Select a generator *g* of group *G* and compute *k* = *g*^*x*^*mod n*. *G* is a multiplicative subgroup of $Z_{n}^{*}$ and order of *G* is *λ*.

**Step 5.** Public key is (*g*,*k*,*n*) and private key is (*p*,*q*,*x*,*x*_*p*_,*x*_*q*_).

Compared to the key generation of rebalanced RSA (Algorithm2.4), only step 4 and 5 are different in Algorithm3.1. Unlike rebalanced RSA, in proposed scheme, modular inverse of private key (i.e., *x*^−1^*mod λ*) is not published and instead, generator *g* of group *G* and *k*(= *g*^*x*^*mod n*) are published.

Encryption and decryption of proposed scheme are identical to CRT–ElGamal (i.e., Algorithm1.2 and 1.3).

### 3.2 Security

In the proposed scheme, RCDH, RDDH and RHDH assumptions are used instead of CDH, DDH and HDH assumptions, respectively. In other words, computational security of proposed scheme is based on the RCDH assumption and semantic security of proposed scheme is based on the RDDH (RHDH) assumption.

In Section 1, we supposed that RDL assumption holds and considered the RCDH, RHDH and RDDH assumptions under the RDL assumption. That is, if the RDL assumption is broken, the one–wayness of *F*(*x*) of Eq (1) is also broken and so, proposed scheme becomes to be insecure.

We mainly considered the upper bound of *x*_*p*_ and *x*_*q*_ that break the RDL assumption in this section.

In the case of rebalanced RSA, only the small CRT exponent attacks have been considered because other attacks except for small CRT exponent attacks are not effective.

Similarly, we considered only the small CRT exponent attacks for the RDL assumption as follows.

**Proposition 1: Let *n* = *pq* where **$p,q,\frac{p-1}{2}$ **and** $\frac{q-1}{2}$ **are primes. If there is a polynomial time algorithm to find the private key (*x*,*x***_***p***_**,*x***_***q***_**,*p*,*q*) from the public key (*g*,*k*,*n*) in proposed scheme when *log***_**2**_***x***_***p***_**≈*log***_**2**_***x***_***q***_**≤*w* for the proper integer** $w(<\frac{1}{2}{log}_{2}n)$**, it is possible to find the private key** (***d*,*d***_***p***_**,*d***_***q***_**,*p*,*q***) **from the public key (*n*,*e*) in rebalanced RSA with full exponent *e*(i.e., *log***_***n***_***e*≈1) when *log***_**2**_***d***_***p***_**≈*log***_**2**_***d***_***q***_**≤*w***.

Proof. In rebalanced RSA, *log*_*n*_*e*≈1 is usually satisfied [17] for *e* such that *ed*≡1 *mod λ* and similarly, *log*_*n*_*e*′≈1 is satisfied for *e*’ such that *xe*′≡1 *mod λ* (i.e., *k*^*e*′^*mod n = g*) in proposed scheme. From the assumption of Proposition1, there exists a polynomial time algorithm (Algorithm*A*) that finds private key (*x*,*x*_*p*_,*x*_*q*_,*p*,*q*) from public key (*g*,*k*,*n*) in proposed scheme.

Hence, by using Algorithm*A*, it is possible to propose the attack algorithm (Algorithm*B*) that breaks the rebalanced RSA with public key (*n*,*e*) satisfying *log*_*n*_*e*≈1 and *log*_2_*d*_*p*_≈*log*_2_*d*_*q*_≤*w* as follows.

**Algorithm B: Attack algorithm to rebalanced RSA which uses Algorithm2.4**.

Input: Public key (*n*,*e*) of rebalanced RSA (*n* = *pq*,*log*_*n*_*e*≈1) such that $\frac{p-1}{2}$ and $\frac{q-1}{2}$ are primes.

Output: Private key (*d*,*d*_*p*_,*d*_*q*_,*p*,*q*)

**Step 1.** Select a generator *m* of *G* with order *λ*, which is a multiplicative subgroup of $Z_{n}^{*}$, and calculate *c*(= *m*^*e*^*mod n*). In this case, *c* also becomes a generator of *G*, because *gcd*(*e*,*λ*) = 1 in rebalanced RSA.

**Step 2**. Search the private key(*x*,*x*_*p*_,*x*_*q*_,*p*,*q*) in polynomial time by using Algorithm *A* after setting *g* = *c* and *k* = *m*. In this case, *m* = *c*^*d*^*mod n*, *d*_*p*_ = *d*_*p*_*mod* (*p*−1), *d*_*q*_ = *d mod* (*q*−1) and *log*_2_*d*_*p*_≈*log*_2_*d*_*q*_≤*w* are satisfied by the assumption and so, it is possible to find private key (*d*,*d*_*p*_,*d*_*q*_,*p*,*q*) in polynomial time.

Of course, unlike proposed scheme, the generator of group *G* is unknown in rebalanced RSA and so, it seems difficult to select *m* as a generator in Step 1 of Algorithm*B*. However, attacker can select the generator without difficulty by selecting a random element $m\in Z_{n}^{*}$ in Step 1, because many elements of $Z_{n}^{*}$ can become the generator of group *G*. Let $p\prime =\frac{p-1}{2}$ and $q\prime =\frac{q-1}{2}$.

From the property of Euler function, the probability that random element *m*∈*Z*_*n*_ becomes a generator of *G* is as follows.

$$\mathrm{Pr}\left[genertor(m)=1|m\in Z_{n}\right]=\frac{\left(p\prime -1)(q\prime -1\right)}{\left(2p\prime +1)(2q\prime +1\right)}=\frac{p\prime q\prime -(p\prime +q\prime )+1}{4p\prime q\prime +2(p\prime +q\prime )+1}\approx \frac{p^{\prime }q^{\prime }}{4p^{\prime }q^{\prime }}=\frac{1}{4}$$

If Algorithm*B* is repeated more than 4 times, then attacker can find private key (*d*,*d*_*p*_,*d*_*q*_,*p*,*q*) by selecting *m* as a generator in Step 1.

From this, rebalanced RSA can be broken in polynomial time. (end of proof.)

Proposition1 shows the rough information for the upper bound of *w* that the small CRT exponent attacks can break the RDL assumption.

The efficient polynomial time attacks to rebalanced RSA (i.e., RSA (250, 250, 2048)) have not been proposed till now [24, 25], even though $p,q,\frac{p-1}{2}$ and $\frac{q-1}{2}$ are primes. Hence, from Proposition1, it is known that the proposed scheme (i.e., CRT–ElGamal (250, 250, 2048)) is also secure from the small CRT exponent attacks. More precisely, Proposition1 and its proof show that if *w* = 250 and modulus is 2048bits number, then $\left(t,\frac{\varepsilon }{4}\right)$ secure RDL assumption holds under the assumption that rebalanced RSA is (*t*,*ε*) secure.

Note. We say that (*t*,*ε*) *RDL* assumption holds in *G* if no *t*-time algorithm has advantage *ε* in solving the RDL problem on *G*. This is the strict definition for the RDL assumption of Section 1 and in this way, the all assumptions of Section 1 and 2 can be redefined more strictly.

In fact, it is not easy to find *e*′(= *x*^−1^*mod λ*) such that *k*^*e*′^*mod n = g*, from the public key (*g*,*k*,*n*) in proposed scheme, because *log*_*n*_*e*′≈1 is usually satisfied and so, finding *e*′ from (*g*,*k*,*n*) becomes a discrete logarithm problem in $Z_{n}^{*}$. (For the composite number *n*, discrete logarithm problem in $Z_{n}^{*}$ is known to be not easier [3, 9, 26] than factoring problem.)

Hence, it is not possible for the attacker to obtain *e*’ and as a result, the lattice based methods and continued fraction methods [14–16, 18–25] cannot be applied to the proposed scheme.

Especially, TLP’s lattice based attack [23–25] which has been known as the state-of-the-art attack to rebalanced RSA cannot be applied to the proposed scheme and so, it can be seen that the proposed scheme is more secure than rebalanced RSA with full exponent *e*(*log*_*n*_*e*≈1).

The practical attack to the proposed scheme can be described as follows.

#### Attacks to the proposed scheme (small private CRT exponent attacks)

From $k_{p}=k\;mod\;p=g^{x_{p}}mod\;p,\;k-k_{p}=k-g^{x_{p}}mod\;p=jp$ is satisfied and so, $\gcd\left(k-g^{x_{p}}mod\;p,pq\right)=p$ is satisfied.

Hence, attack to the proposed scheme is finalized as finding *i* that satisfies

$$gcd\;(k-g^{i}mod\;n,n)\neq 1$$

for all available *i* because CRT exponents are small.

Thus, attack of [17] to rebalanced RSA is straightly applicable to the proposed scheme as follows.

**Proposition 2: Let *n* = *pq* where** $p,q,\frac{p-1}{2}$ and $\frac{q-1}{2}$ **are primes,** $\lambda =\frac{\left(p-1)(q-1\right)}{2},$ ***x***_***p***_ = ***x mod*** (***p***−**1**) **and *x***_***q***_ = ***x mod*** (***q*−1) with *x***_***p***_**<*x***_***q***_ **for the random selected number** $x\in Z_{n}^{*}$.

**Then modulus *n* can be factored in time** $\left(x_{p}^{1/2}logx_{p}\right)$ **by using generator *g* of subgroup of** $Z_{n}^{*}$ **with order *λ* and *z* = *g***^***x***^***mod n***.

(Proof): The proof is identical to the case of rebalanced RSA, which has been explained in detail in [17], and so skipped here. (end of Proof).

Judging with $O(x_{p}^{1/2}logx_{p})$, CRT-exponents *x*_*p*_ and *x*_*q*_ should be at least 160bits long for 1024bits modulus and at least 224bits for 2048bits modulus in order for this attack to match the current estimated complexity of factoring the modulus for those sizes [17].

Unlike Proposition1, Proposition2 shows the more exact information for the upper bound of *w* that the small CRT exponent attacks can break the RDL assumption. That is, Proposition2 and its proof show that if *w* = 224 and modulus is 2048bits number, then (*t*,*ε*) secure RDL assumption holds.

There are two methods to break the RCDH assumption which is the basis of the computational security of proposed scheme. One is to break the DL assumption and the other is to break RDL assumption. RDL assumption is weaker than DL assumption, which is known to be hard [7–9], and so, breaking RDL becomes best known method to break RCDH.

From the intractability for breaking RDL assumption mentioned above, it can be seen that CRT–ElGamal (224, 224, 2048) (i.e., proposed scheme) is computationally (*t*,*ε*) secure. In other words, (*t*,*ε*) RCDH assumption holds when *w* = 224 for the 2048 bits modulus.

Hence, if *H*(*G*^2^→*K*_*s*_) is modeled as a random oracle, proposed scheme becomes to be semantically secure.

Next, we considered the RDDH and RHDH assumptions under the RCDH assumption in order to prove the semantic security of proposed scheme without random oracle.

Under the RDL and RCDH assumptions, we can simply prove that DDL, RHDH and RDDH assumptions hold in the similar way to [4], which described the DDH with short exponents under the DLSE assumption. That is, the following Proposition 3 (DDL) and Proposition 4 (RHDH) can be easily obtained by substituting RDL assumption for DLSE [4, Assumption4] in Proposition1 and Theorem5 of [4].

**Proposition 3: Let *n* = *pq* where** $p,q,\frac{p-1}{2}$ **and** $\frac{q-1}{2}$ **are primes,** $\lambda =\frac{\left(p-1)(q-1\right)}{2}$ **and *G* is a subgroup of** $Z_{n}^{*}$ **of order *λ*. If RDL assumption holds, then DDL assumption holds in *G***.

(Proof): In [7], Patel and Sundaram introduced the concept of HB (Hardness of Bits) connected to the one-way function and first described the HB under the *s*-DLSE (Discrete Logarithm with Short *s*-Bit Exponent) assumption. Referring to the result of [7], Gennaro [4, 8] introduced the concept of SEI (Short-Exponent Indistinguishability) and described the difficulty of SEI (i.e., SEI assumption) under the *s*-DLSE assumption. More exactly, SEI assumption in $Z_{p(=2q+1)}^{*}$ where both *p* and *q* are primes was proved in [8] and generalization of this result (i.e., SEI assumption for the cyclic group *G* with order *ord*(*G*), such that *ord*(*G*) is odd or $\frac{ord(G)}{2}$ is odd) was introduced in Proposition1 of [4].

From the fact above, SEI assumption holds under the DLSE assumption in *G*, because *λ*, which is the order of *G*, is even, but *λ*/2 is odd.

Meanwhile, the DLSE assumption and RDL assumption are identical in the aspect that they are all based on intractability of cracking the discrete logarithm when restricted exponents are used instead of full exponents. (In the case of *s*-DLSE, *s*-bits exponent is used instead of full exponent and in the case of *w*-RDL, *w*-bits CRT exponents are used instead of full CRT exponents. The relation between *s*-DLSE and *w*-RDL is described in detail in the proof of Proposition4.)

From this, RDL can be used instead of DLSE in the proof of SEI assumption in *G* and as a result, the proof of DDL is derived. That is, the proof of DDL can be easily obtained by only substituting RDL for DLSE in the proof of SEI assumption [4, Proposition1], which was described in detail in Appendix C of e-print version of [4] and so, skipped here. (end of Proof).

**Proposition 4: Let *n* = *pq* where** $p,q,\frac{p-1}{2}$ **and** $\frac{q-1}{2}$ **are primes,** $\lambda =\frac{\left(p-1)(q-1\right)}{2}$ **and *G* is a subgroup of** $Z_{n}^{*}$ **of order *λ*. If RDL assumption holds, then RHDH assumption also holds in *G***.

(Proof): In [4], Gennaro proposed *t*-DDH assumption as a relaxation of a DDH assumption and described the possibility of secure DH key transform by hash function in the non-DDH group.

Note. 0≤*t*≤*log* (*ord*) is satisfied and DDH assumption can be seen a special case (*t* = *log*(*ord*)) of *t*-DDH assumption [4] when *ord* denotes the order of group.

Besides, Gennaro proved that if *s*-DLSE and *t*-DDH assumptions hold, then hashed DH transform is as secure with full exponents as with *s*-bit exponents (i.e., HDH assumption with short exponents holds) in non-DDH group [4, Theorem5].

From the fact above, HDH assumption with short exponents holds under the DLSE assumption in *G*, because *G* is a DDH group [3] (i.e., *log* (*ord*)-DDH assumption holds in *G*).

Then, what is the relation between DLSE and RDL assumptions in group *G*?

As in the other groups, square-root attacks such as Shanks and Pollard methods are usually used in order to break the DLSE assumption in *G*, too. And, unlike the other groups, small CRT exponent attacks can be additionally used for breaking DLSE in *G*. However, the best known small CRT exponent attack is also square-root algorithm (mentioned in Proposition2) and so, the best known attack for DLSE still remains the square-root attack. That is, 224-DLSE assumption still holds for the 2048 bits modulus in *G*, even if the small CRT exponent attacks are considered.

From the fact above, it seems that DLSE and RDL assumption are quite identical in security. However, RDL assumption is strictly stronger (i.e., *DLSE*⟸*RDL*) than DLSE assumption in the aspect that, in order to break the RDL, Shanks and Pollard methods cannot be used and only small CRT exponent attack can be used.

From this, RDL can be used instead of DLSE in the proof of HDH assumption with short exponents in *G* and as a result, the proof of RHDH can be obtained. The proof of RHDH is identical to the proof of HDH with short exponents [4, Theorem5] except that RDL is used instead of DLSE and so, skipped here. (end of Proof).

Finally, let’s consider the RDDH assumption. In [4], on the basis of Theorem5, Gennaro described that performing the DH transform with exponent of size *s* yields values which are indistinguishable from random element in DDH-group where *s*-DLSE assumption holds (i.e., described that DDH assumption with short exponents holds under the DLSE and DDH assumption).

As mentioned in the proof of Proposition4, DDH and DLSE assumptions hold in the subgroup *G* of $Z_{n(=pq)}^{*}$ of order $\lambda (=\frac{\left(p-1)(q-1\right)}{2})$ where $p,q,\frac{p-1}{2}$ and $\frac{q-1}{2}$ are primes (i.e., *G* is a DDH group in which DLSE assumption holds) and so, DDH assumption with short exponents also holds in *G*.

Similarly to Proposition3 and 4, DLSE can be replaced with RDL in the proof of DDH assumption with short exponents, too. As a result, following Proposition5 (i.e., the proof of RDDH in *G*) can be easily obtained.

**Proposition 5: Let *n* = *pq* where *p*,*q*,**$\frac{p-1}{2}$ **and** $\frac{q-1}{2}$ **are primes**, $\lambda =\frac{\left(p-1)(q-1\right)}{2}$ **and *G* is a subgroup of** $Z_{n}^{*}$ **of order *λ*. If RDL assumption holds, then RDDH assumption holds in *G***.

From Proposition4 and 5, it is known that proposed scheme becomes to be semantically secure even if *H*(*G*^2^→*K*_*s*_) is not modeled as a random oracle.

From the all facts above, it is proved that regardless of what security properties the hash function *H* may possess, proposed scheme (i.e., CRT–ElGamal (224, 224, 2048) with/without random oracle) is semantically secure.

## 4. Performance comparison

We have used the hybrid encryption paradigm for the performance analysis. (See Algorithm 1.2, 1.3, 2.2 and 2.3.) In comparison, all public key schemes have been used as KEM (Key Encapsulation Mechanism) and the same symmetric authentication encryption (*E*_*s*_,*D*_*s*_) has been used as DEM (Data Encapsulation Mechanism) for all public key schemes.

In this case, delays by hash function and symmetric authenticated encryption can be ignored compared to modular exponentiation of big integers and so, we have considered only the encryption and decryption times of public key schemes in the comparison.

### 4.1 Theoretical results

The theoretical encryption and decryption time comparison of CRT–RSA, rebalanced RSA, ElGamal in subgroup of quadratic residues in $Z_{p(=2p^{\prime }+1)}^{*}$ where both *p* and *p*′ are primes (noted as ElGamal), ElGamal in subgroup of $Z_{p(=aq+1,a>2)}^{*}$ of order *q* where both *p* and *q* are primes (noted as ElGamal in subgroup), CRT–ElGamal, ElGamal with short exponents (noted as SE–ElGamal) and proposed scheme are summarized in Table 1.

**Table 1:** Theoretical encryption and decryption time comparison.

	CRT-RSA	Rebalanced RSA	ElGamal	ElGamal in subgroup	SE-ElGamal	CRT-ElGamal	Our scheme
**Encryption Exponent (Hamming Weight)**	17 bits (2)	2048 bits (1024)	2047 bits (32)	224 bits (112)	2048 bits (32)	2048 bits (32)	2048 bits (32)
**Number of Multiplication in Encryption**	16+1 = 17	2048×1.5 = 3072	32×2 = 64	112×2 = 224	32×2 = 64	32×2 = 64	32×2 = 64
**Unit Time for Encryption**	0.0056	1	0.02	0.073	0.02	0.02	0.02
**Decryption Exponent**	2048 bits	2048 bits	2047 bits	224 bits	224 bits	2048 bits	2048 bits
**CRT Exponent**	1024 bits	250 bits	–	–	–	1024 bits	224 bits
**Number of Multiplication in Decryption (Modular Size)**	2×1024×1.5 +2 = 3074 (1024)	2×250×1.5+2 = 752 (1024)	2047×1.5 = 3070.5 (2048)	224×1.5 = 336 (2048)	224×1.5 = 336 (2048)	2×1024×1.5 +2 = 3074 (1024)	2×224×1.5+2 = 674 (1024)
**Unit Time for Decryption**	0.25	0.061	1	0.109	0.109	0.25	0.055
**Total Processing Time Max (Encryption, Decryption)**	0.25	1	1	0.109	0.109	0.25	0.055

**Note**. The comparison has been done with the numbers of executed exponentiations under the assumption that a full modular exponentiation (i.e., *g*^*x*^ *mod n*, where both *x* and *n* are the 2048-bit numbers) takes one unit time.

In the encryption of ElGamal type schemes of Table 1, for the fast encryption, pre–calculated table that contains the main exponentiations of generator and public key were used. And the random exponents with Hamming weight 32 were used (ElGamal in subgroup of $Z_{p}^{*}$ of order *q*(≈2^224^) is an exception) for the 2048bits modulus, because the possible number of exponents is large enough (i.e., $C_{2048}^{32}>C_{2047}^{32}>2^{224}$ and $C_{2048}^{16}>C_{2047}^{16}>2^{112}$ are satisfied) to preclude known attacks [9, Note 3.59, Note 8.21].

Referring to Section 3.2, [17, Section 4], [23] and [24], 224 bits and 250bits CRT exponents were used in decryption of proposed scheme and rebalanced RSA, respectively.

And, referring to [4, Section 4], [6] and [9, Section 3.6], 224 bits private exponent was used in decryption of SE–ElGamal and ElGamal in subgroup to thwart the usual square–root attacks such as Shanks and Pollard methods.

As shown in Table 1, proposed scheme is more advantageous than other systems because both encryption and decryption are fast. In theory, the total processing speed of proposed scheme is approximately 18.2 times faster than ElGamal (or rebalanced RSA), 4.54 times faster than CRT–RSA (or CRT–ElGamal) and 2 times faster than SE–ElGamal (or ElGamal in subgroup), respectively.

## 4.2 Experimental results

The experimental results for the schemes of Table 1 are listed in Table 2, which shows the relative comparison between the schemes, because the timings are approximate.

**Table 2:** Practical execution time comparison.

	CRT-RSA	Rebalanced RSA	ElGamal	ElGamal in subgroup	SE-ElGamal	CRT-ElGamal	Our scheme
**Encryption**	0.81	122	2.61	9.3	2.61	2.61	2.61
**Decryption**	31	7.9	122	13.7	13.7	31	7.1
**Total Processing Time Max (Encryption, Decryption)**	31	122	122	13.7	13.7	31	7.1

**Note**. The timings were made on a 3.5GHz Core i3-4150 desktop using NTL with GMP Library and were all in ms.

The prime generation module to find a prime *p* = 2*p*′+1 where *p*′ is a prime number was used in all schemes of Table 2 (ElGamal in subgroup is an exception).

In fact, as shown in Table 2, the total processing speed of CRT–ElGamal is the same as the one of CRT–RSA, but there exists a message expansion by factor of 2 compared to CRT–RSA and so, CRT–ElGamal is of no practical use compared to CRT–RSA. However, in the case of reducing the CRT exponents, CRT–ElGamal is more advantageous than CRT–RSA in total processing speed, because encryption time of CRT–ElGamal is not affected by reducing CRT exponents unlike CRT–RSA. (Compare the rebalanced RSA with proposed scheme of Table 2).

For the 2048 bits modulus, proposed scheme is almost 4.37 times faster than CRT–RSA and 1.93 times faster than SE–ElGamal (or ElGamal in subgroup) in total encryption processing.

## 5. Discussion

In Section 3.2, we proved that CRT–ElGamal (224, 224, 2048) is still one-way (i.e., RCDH assumption holds) under the RDL and CDH assumptions and is still IND-CPA (i.e., RHDH and RDDH assumption hold) under the RDL and DDH assumptions. In other words, Section 3 described that RDL assumption can be used for the CPA security.

However, RDL can also be used for the CCA security when it is used in CCA secure encryption schemes such as twin ElGamal and Cramer-Shoup scheme. That is, twin ElGamal and Cramer-Shoup scheme can be modified for the fast decryption in the similar way to Section 3. In this case, our variant of twin ElGamal becomes to be IND-CCA under the RCDH assumption in the random oracle model and our variant of Cramer-Shoup becomes to be IND-CCA under the RDDH assumption. The security proof can be easily obtained by substituting RCDH and RDDH for CDH and DDH, respectively, in the security proof of ordinary twin ElGamal and Cramer-Shoup scheme.

Finally, the DLSE assumption can be used for both DH and ElGamal protocol, but RDL assumption can be used for only the ElGamal protocol.

## 6. Conclusion

In this paper, we did not suggest any new encryption protocols. We described only the possibility of reducing CRT exponents in CRT–ElGamal which is known to be semantically secure under the combination with hash function and symmetric authenticated encryption.

In other words, we considered RCDH, RDDH and RHDH assumptions and described that these assumptions can be substituted for CDH, DDH and HDH assumptions, respectively, in protocols. By using such substitutions, we achieved the decryption speed up without losing of fast encryption in CRT-ElGamal (with/without random oracle).

Total processing speed of the proposed scheme is comparable to the decryption speed of rebalanced RSA. Hence, the proposed scheme is suited for the applications which require the fast speed in both encryption and decryption.

Substitutions above give the possibility of speed up in some CCA secure ElGamal protocols, too. Especially, it would be possible to propose the fast variant of twin ElGamal which has the fast encryption and fast decryption by using RCDH assumption instead of CDH assumption.
